# Comparison between atlas and convolutional neural network based automatic segmentation of multiple organs at risk in non-small cell lung cancer

**DOI:** 10.1097/MD.0000000000021800

**Published:** 2020-08-21

**Authors:** Tao Zhang, Yin Yang, Jingbo Wang, Kuo Men, Xin Wang, Lei Deng, Nan Bi

**Affiliations:** Department of Radiation Oncology, National Cancer Center/National Clinical Research Center for Cancer/Cancer Hospital, Chinese Academy of Medical Science and Peking Union Medical College, Beijing, China.

**Keywords:** automatic segmentation based on atlas, automatic segmentation based on convolutional neural network, non-small cell lung cancer, organs at risk, postoperative radiation therapy

## Abstract

Delineation of organs at risk (OARs) is important but time consuming for radiotherapy planning. Automatic segmentation of OARs based on convolutional neural network (CNN) has been established for lung cancer patients at our institution. The aim of this study is to compare automatic segmentation based on CNN (AS-CNN) with automatic segmentation based on atlas (AS-Atlas) in terms of the efficiency and accuracy of OARs contouring.

The OARs, including the lungs, esophagus, heart, liver, and spinal cord, of 19 non-small cell lung cancer patients were delineated using three methods: AS-CNN, AS-Atlas in the Pinnacle^3^-software, and manual delineation (MD) by a senior radiation oncologist. MD was used as the ground-truth reference, and the segmentation efficiency was evaluated by the time spent per patient. The accuracy was evaluated using the Mean surface distance (MSD) and Dice similarity coefficient (DSC). The paired t-test or Wilcoxon signed-rank test was used to compare these indexes between the 2 automatic segmentation models.

In the 19 testing cases, both AS-CNN and AS-Atlas saved substantial time compared with MD. AS-CNN was more efficient than AS-Atlas (1.6 min vs 2.4 min, *P* < .001). In terms of the accuracy, AS-CNN performed well in the esophagus, with a DSC of 73.2%. AS-CNN was better than AS-Atlas in segmenting the left lung (DSC: 94.8% vs 93.2%, *P* = .01; MSD: 1.10 cm vs 1.73 cm, *P* < .001) and heart (DSC: 89.3% vs 85.8%, *P* = .05; MSD: 1.65 cm vs 3.66 cm, *P* < .001). Furthermore, AS-CNN exhibited superior performance in segmenting the liver (DSC: 93.7% vs 93.6%, *P* = .81; MSD: 2.03 cm VS 2.11 cm, *P* = .66). The results obtained from AS-CNN and AS-Atlas were similar in segmenting the right lung. However, the performance of AS-CNN in the spinal cord was inferior to that of AS-Atlas (DSC: 82.1% vs 86.8%, *P* = .01; MSD: 0.87 cm vs 0.66 cm, *P* = .01).

Our study demonstrated that AS-CNN significantly reduced the contouring time and outperformed AS-Atlas in most cases. AS-CNN can potentially be used for OARs segmentation in patients with pathological N2 (pN2) non-small cell lung cancer.

## 1 Introduction

Lung cancer is the leading cause of cancer-related death not only in China but also around the world,^[1]^ with non-small cell lung cancer (NSCLC) constituting 85% of the total cases. Surgery is the first choice for patients diagnosed with NSCLC. Postoperative radiation therapy (PORT) is a necessary consideration for patients with operable pathological N2 (pN2) NSCLC,^[2]^ as it has been demonstrated to decrease local recurrence and improve survival significantly.^[3–5]^

Radiotherapy technology has witnessed tremendous advancements in recent years, with the increasing utilization of intensity modulated radiation therapy,^[6]^ volumetric modulated arc therapy,^[7]^ image guided radiation therapy^[8]^ and proton therapy.^[9]^ The main goals of modern radiation therapy (RT) are to maximize tumor control and minimize RT-related toxicity, which are achieved by delivering curative doses to tumor targets while sparing the irradiation of organs at risk (OARs). However, optimizing the delivery of reduced dose to normal tissues requires accurate delineation of the interest (ROI). As a result, accurate OARs contouring is of vital importance to RT planning, which is closely related to the quality and outcome in lung cancer care.

OARs Delineation is a tedious and time-consuming process for the radiation oncologist. Furthermore, the quality of delineation depends on the expertise level of the individual observer. Even though they delineated ROI according to the radiation therapy oncology group (RTOG) guidelines, considerable inter-observer and intra-observer variations still exist.^[10,11]^ Automatic segmentation software has the potential to improve efficiency, accuracy, and consistency between observers.

Software of automatic segmentation based on atlas (AS-Atlas) has been commonly used in radiotherapy treatment planning,^[12–14]^ which applies deformable image registration methods to propagate the labeled structures in the atlas image onto the target image automatically.^[15]^ However, two time-consuming procedures of dividing patients into different groups according to their size and registering the computed tomography (CT) images to their atlas images have limited the use of AS-Atlas in clinical practice.

Recently, automatic segmentation based on convolutional neural network (AS-CNN) has gained popularity in RT.^[16,17]^ AS-CNN can discover the informative representations in a self-taught manner, use hierarchical layers to learn abstraction, and accomplish high-level tasks efficiently. However, a comparison between AS-CNN and AS-Atlas for RT has been rarely reported. In the case of lung cancer, only one study has compared the automatic segmentation of OARs between AS-CNN and AS-Atlas.^[18]^

Our group has previously established several AS-CNN models for target segmentation or OARs segmentation in rectal,^[19]^ nasopharyngeal,^[20]^ and breast^[15]^ cancers. In this study, the AS-CNN model is used for the segmentation of OARs in lung cancer. Concurrently, the new AS-CNN model is compared with the AS-Atlas contour in specific patients with pN2 NSCLC, who can gain more benefit from PORT among all stages of NSCLC. The aim of this study is to improve the utility of AS-CNN in clinical practice to relieve the radiation oncologists of the labor-intensive contouring work and increase the accuracy, consistency and efficiency of ROI segmentation.

## 2 Patients and methods

### 2.1 Patient selection

A total of 250 patients with pN2 NSCLC who received PORT after R0 resection from January 2005 to December 2014 at our department were retrospectively selected to build the AS-CNN model, of which 200 cases were randomly assigned to the training set and 50 cases to the validation set. An additional 19 patients diagnosed with NSCLC from December 2016 to July 2018 at our department were selected as the testing group for the automatic delineation of OARs. The Ethics Committee of Cancer Institute and Hospital Board Affiliation of Chinese Academy of Medical Sciences approved the study, and all patients provided informed consent before enrollment. All patients were simulated at the supine position with both forearms crossed above the forehead. Simulation contrast computed CT data were acquired on a Somatom Definition AS 40 (Siemens Healthcare, Best, the Netherlands) system set to the helical scan mode. The CT images were reconstructed using a matrix size of 512 × 512 and thickness of 5.0 mm. The OARs, including the left lung, right lung, heart, spinal cord, esophagus, and liver, were delineated by multiple experimental radiation oncologists of our department with specialization in the thoracic region.

### 2.2 AS-CNN model for segmentation

In this study, an AS-CNN model based on ResNet-101^[21]^ is introduced for segmenting the OARs in PORT patients. The dilated model could extract original information from the CT images by introducing different dilation factors. The input to deep AS-CNN model was 2D CT images and the output was the corresponding labels of the OARs. A total of 200 randomly selected thoracic and abdominal CT images, including manual segmentation labels of the OARs, were used as the training test to adjust the parameters of the network. The remaining50 cases were assigned to the validation set to test the stability of the AS-CNN model.

### 2.3 Contour methods

Each of 19 patients in the testing group was delineated into 3 sets of OARs using manual delineation (MD), AS-CNN and AS-Atlas respectively. All manual contours were drawn on a software used in clinical practice (Pinnacle^3^software, Version: 9.10, Fitchburg, WI) by a single radiation oncologist specialized in the thoracic region according to the radiation therapy oncology group guidelines to prevent any inter-observer variability. All atlas-based contours were automatically segmented using the commercial AS-Atlas software in Pinnacle. To obtain the AS-CNN contours, the observers imported the CT images to the CNN model established by our group, and then the OARs were automatically generated. The time spent per patient was recorded, with an accuracy of the seconds.

### 2.4 Accuracy assessment

The performances of the proposed two automatic methods were evaluated in terms of the Dice similarity coefficient (DSC) and Mean surface distance (MSD), MD was used as the ground-truth reference.

The DSC is defined as: 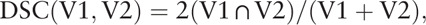


where V1 denotes the volume of manually delineated organ, and V2 represents the volume of the automatically segmented organ. V1∩V2 corresponds to the common volume between V1 and V2. The DSC value ranges between 0 and 1 (0 = no overlap, 1 = complete overlap).

The MSD is the mean distance between the surfaces of two volumes. As the MSD decreases, the overlap between two volumes increases.

### 2.5 Statistical analysis

To evaluate the DSC, MSD, and delineation time between different contouring methods, the pared t-test or Wilcoxon signed-rank test was performed according to the normality of the data. These analyses were all performed using the SPSS version 23.0 software (SPSS, Inc. IBM, Armonk, NY). A value of *P* < .05 was considered statistically significant.

## 3 Results

### 3.1 Efficiency

For the chest and abdomen CT images of 19 cases in the testing set, the average time acquired for MD was 25.4 minutes, whereas the average time required by AS-CNN and AS-Atlas was 1.6 minutes and 2.4 minutes, which accounted for only 7% and 10% of time spent on MD, respectively. AS-CNN was more efficient than AS-Atlas (*P* < .001, Wilcoxon signed-rank test).

### 3.2 Accuracy

**Figure 1:**
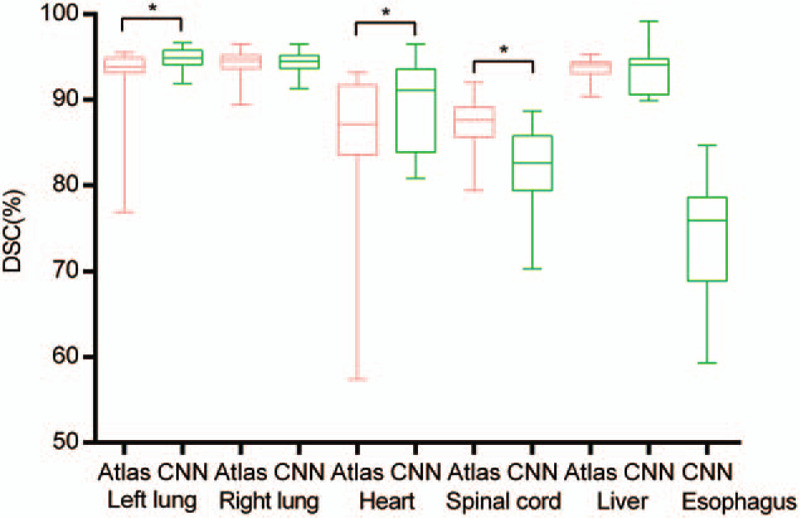
DSCs of the AS-CNN and AS-Atlas, with manual contours as the standard/reference. The pink lines correspond to the results from AS-Atlas, the green lines correspond to those from AS-CNN. ^∗^indicates a significant difference (*P* < .05).

**Figure 2:**
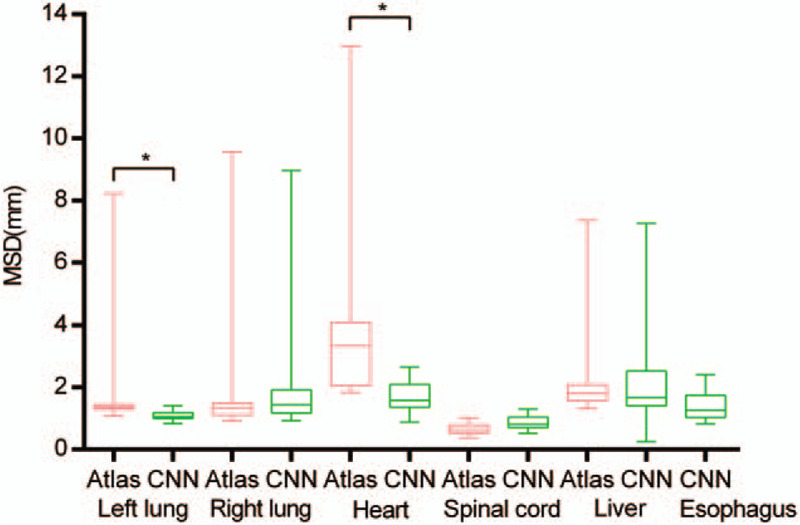
MSDs of AS-CNN and AS-Atlas, with manual contours as the standard. The pink lines correspond to the results from AS-Atlas, the green lines correspond to the results from AS-CNN. ^∗^indicates a significant difference (*P* < .05).

**Figure 3:**
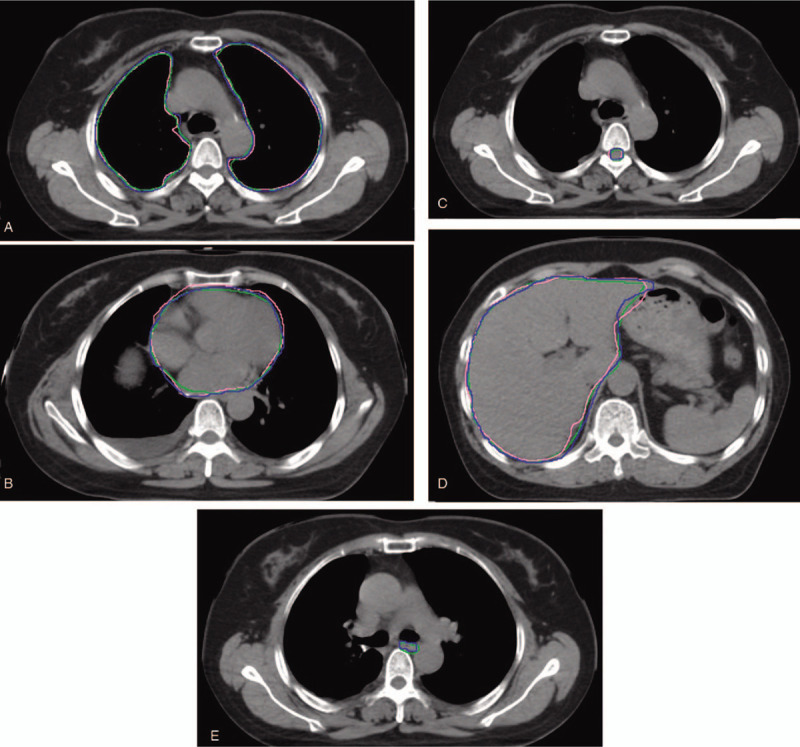
Example cases showing MD (blue line), AS-Atlas (pink line), and AS-CNN (green line) for the lung, heart, spinal cord, liver, and esophagus.

The DSC values are listed in Table 1 and Figure 1. The MSD values are listed in Table 1 and Figure 2. AS-CNN performed well in segmenting the esophagus, with an average DSC value of 73.2%, while that of the AS-Atlas for the esophagus was unavailable. AS-CNN performed better in segmenting the left lung (DSC: 94.8% vs 93.2%, *P* = .01, Wilcoxon signed-rank test; MSD: 1.10 cm vs 1.73 cm, *P* < .001, Wilcoxon signed-rank test) and heart (DSC: 89.3% vs 85.8%, *P* = .05, Wilcoxon signed-rank test; MSD: 1.65 cm vs 3.66 cm, *P* < .001, Wilcoxon signed-rank test). In addition, AS-CNN exhibited a trend of superior performance in segmenting the liver compared with AS-Atlas (DSC: 93.7% vs 93.6%, *P* = .81, pared *t* test; MSD: 2.03 cm vs 2.11 cm, *P* = .66, paired *t* test). The results obtained from AS-CNN and AS-Atlas were similar for the contouring of the right lung (DSC: 94.3% vs 94.3%, *P* = .44, Wilcoxon signed-rank test; MSD: 2.23 cm vs 2.17 cm, *P* = .31, paired *t* test). However, the performance of AS-CNN for the spinal cord was inferior compared with that of AS-Atlas (DSC: 82.1% vs 86.8%, *P* = .01, Wilcoxon signed-rank test; MSD: 0.87 cm vs 0.66 cm, *P* = .01, paired *t* test). The contours of one representative case generated by the 3 segmentation models are shown in Figure 3.

**Table 1:**
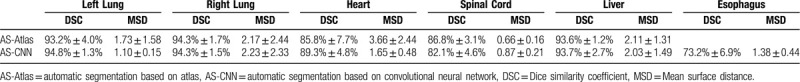
The Dice similarity coefficient and Mean surface distance results for all organs at risk.

### 3.3 Contour volume

**Figure 4:**
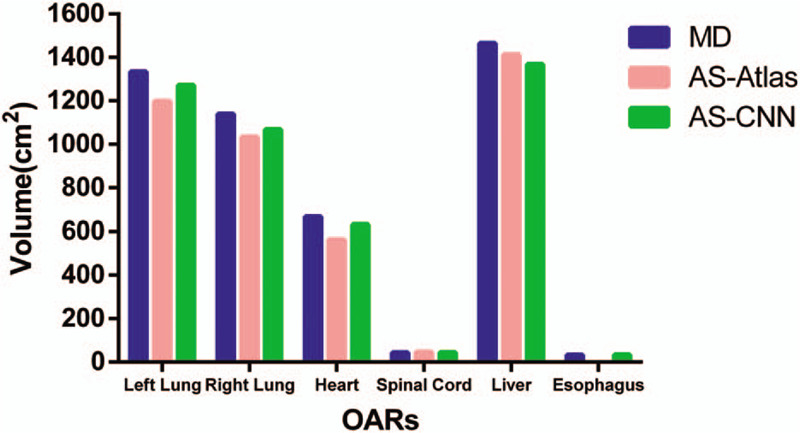
Comparison between the average Volumes of all OARs segmented by 3 models.

The average volumes of all OARs, including the left lung, right lung, heart, spinal cord, liver, and esophagus, produced by MD, AS-Atlas, and AS-CNN, are listed in Figure 4 and Table 2. The average volumes of the liver, left lung, right lung and heart were greater than 500 cm^3^ whereas those of the spinal cord and esophagus were less than 100 cm^3^. The average volumes of 4 organs, namely the left lung, right lung heart and spinal cord segmented by AS-CNN were comparable to the volumes contoured by MD. In contrast, the average volume of one organ, namely the liver, segmented by AS-Atlas was comparable to the volume delineated by MD.

**Table 2:**
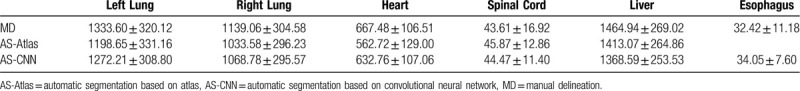
The average volumes of all organs at risk segmented using 3 methods.

## 4 Discussion

In this study, the OARs segmented by AS-CNN and AS-Atlas were compared. Following training and validation, the AS-CNN can be used to segment normal organs in the 2D CT images accurately and efficiently. Zijdenbos et al suggested that a DSC value >70% represented good overlap.^[22]^ In this study, the average DSC values of AS-CNN were 94.8%, 94.3%, 89.3%, 93.7%, 82.1%, and 73.2% for the left lung, right lung, heart, liver, spinal cord, and esophagus, respectively. All OARs had DSC values above 70%, suggesting that AS-CNN achieved high accuracy in OARs segmentation.

Among the 6 organs, the left and right lungs had the highest DSC values of 94.8%, and 94.3% respectively. This superior performance can be attributed to the large size and high soft tissue contrast of the lungs in the CT images.

Compared with AS-Atlas, AS-CNN had an additional function for contouring the esophagus that was not available in AS-Atlas. In addition, AS-CNN performed significantly better in the segmentation of the left lung (DSC 94.8% vs 93.2%; MSD 1.10 cm vs 1.73 cm) and heart (DSC 89.3% vs 85.8%; MSD 1.65 cm vs 3.66 cm) compared with AS-Atlas. AS-CNN exhibited superior trends for the DSC (93.7% vs 93.6%) and MSD (2.03 cm vs 2.11 cm) in the liver segmentation. These data suggest that the proposed AS-CNN outperformed AS-Atlas in most cases of OARs delineation for lung cancer. However, in the segmentation of the spinal cord, AS-CNN underperformed compared with AS-Atlas, indicating that there might be room for further improvement, and adjustments to AS-CNN might be required. For the automatic segmentation of the spinal cord, DSC values of 0.8,^[23]^ 0.71,^[24]^ 0.74,^[25]^ and 0.76^[18]^ have been reported in other literatures. The AS-CNN performed better in segmenting the spinal cord compared with models mentioned in other studies.

Other groups have also established various automatic segmentation models to contour OARs. Schreibmann et al^[25]^ assessed the performance of their multi-atlas segmentation software. They reported an average DSC of 0.958, 0.912, and 0.740 for the lung, liver, and spinal cord, respectively. Zhu et al^[18]^ compared their deep convolutional neural network-based technique with atlas-based technique. They reported an average DSC of 0.95, 0.91, 0.89, 0.76, and 0.64 for the lung, heart, liver, spinal cord, and esophagus, respectively. Here, our AS-CNN approach performed better in the heart, spinal cord, esophagus, and liver, and showed similar performance for the lung compared with other models from published literatures.

The DSC value has been generally used to evaluate the efficiency of automatic ROI segmentation, whereas the MSD has been rarely reported in other studies. Zhu et al^[18]^ reported an average MSD of 1.93, 3.21, 2.92, 1.81, and 2.65 for the lung, liver, heart, spinal cord, and esophagus, respectively. However, in our study, the average MSD is 1.10, 2.23, 2.03, 1.65, 0.87, and 1.38 for the left lung, right lung, liver, heart, spinal cord, and esophagus. Here, our AS-CNN model showed superior performance in all OARs segmentations compared with other published models.

For the 19 test cases, the average time taken for delineating the six organs by MD was 25.4 minutes, whereas the average time required by AS-CNN was 1.6 minutes, which only accounted for 7% of the MD time. Compared with AS-Atlas that required an average time of 2.4 minutes, AS-CNN was more efficient (*P* < .001). This was because the process of transforming the CT images into atlas images consumed considerable time in AS-Atlas,^[15]^ whereas the trained AS-CNN could quickly segment the ROI without requiring any changes to the CT images.

This work was not pioneering with regard to using a CNN model to segment organs in lung cancer. However, this work was the first attempt to apply CNN to for the segmentation of OARs in patients with operable pN2 NSCLC, who could gain more benefit during PORT. A comparing between the performances of AS-CNN and AS-Atlas revealed that AS-CNN was more efficient and accurate in contouring organs. AS-CNN can potentially be used in clinical practice to relieve radiation oncologists of the labor-intensive contouring work. However, the segmentation accuracy can be further improved by increasing the amount of training and validation data, optimizing the AS-CNN algorithm, replacing the CT images with MRI images with high soft tissue contrast, and combining AS-CNN and AS-Atlas.

## 5 Conclusion

In this study, an AS-CNN model was established for segmenting six OARs in chest and abdominal CT images of patients with pN2 NSCLC. In addition, the performance of AS-CNN was compared with AS-Atlas. The results demonstrated that the proposed AS-CNN outperformed AS-Atlas in most cases. Therefore, AS-CNN has the potential to segment OARs in patients with pN2 NSCLC who require PORT.

## Author contributions

**Conceptualization:** Tao Zhang, Nan Bi

**Data curation:** Yin Yang, Jingbo Wang, Kuo Men

**Formal analysis:** Tao Zhang, Yin Yang

**Resources:** Tao Zhang, Xin Wang, Lei Deng

**Software:** Tao Zhang, Kuo Men, Nan Bi

**Writing – original draft:** Yin Yang, Tao Zhang

**Writing – review & editing:** Tao Zhang, Nan Bi
